# Cost-effectiveness and budget impact analyses of strategies to improve measles vaccination coverage in Kenya

**DOI:** 10.1371/journal.pgph.0006440

**Published:** 2026-09-22

**Authors:** Stacey Orangi, Prashant Mandaliya, Rose Jalang’o, Patrick Amoth, Alick Banda, Chibueze Anosike, Felix Masiye, Obinna Onwujekwe, Edwine Barasa

**Affiliations:** 1 Health Economics Research Unit, KEMRI-Wellcome Trust Research Programme, Nairobi, Kenya; 2 Ministry of Health, Government of Kenya, Nairobi, Kenya; 3 Department of Economics, University of Zambia, Lusaka, Zambia; 4 Health Policy Research Group, University of Nigeria, Nsukka, Nigeria; 5 Centre for Tropical Medicine and Global Health, Nuffield Department of Medicine, University of Oxford, Oxford, United Kingdom; PLOS: Public Library of Science, UNITED STATES OF AMERICA

## Abstract

Measles control efforts have been implemented over the years. However, elimination of the disease is yet to be realized. Additionally, global measles vaccination coverage has stagnated over the past decade, leading to outbreaks. We assessed the cost-effectiveness and budget impact of scaling-up vaccination coverage in Kenya, towards the measles elimination goal. A cost-effectiveness analysis was conducted using a Markov model from a health system’s perspective over a 10-year time frame, incorporating a new birth cohort annually. Two vaccination scenarios were examined 1) a constant coverage improvement scenario, assuming current funding is maintained resulting in annual increases of 0.3% and 2% in the first and second-dose measles vaccine coverage, respectively, alongside supplementary immunization activities(SIAs) and 2) an intensified investment scenario, assuming achievement of 95% coverage for both vaccine doses in addition to SIAs. The later assumed increased investments to reach hard-to-reach populations. A threshold of USD 1,280 per disability adjusted life year (DALY) averted was applied. A budget impact analysis was also conducted. Over a 10-year time horizon, the constant coverage improvement scenario led to 6% fewer deaths (213 (161–276)) and was cost-effective (incremental cost-effectiveness ratio, ICER = USD 1,269 ($1,205-$1,333) per DALY averted) compared to the base scenario. Increasing coverage in the intensified investment scenario, further reduced deaths by 25% (879(626--1,284)) and was more cost effective, ICER 1,093 (1,029 -1,157) per DALY averted, compared to the constant improvement scenario. The constant improvement scenario had a moderate budget impact while the intensified improvement scenario had a very high budget impact, when assessed against Kenya’s health expenditure. Our findings suggest that accelerating measles coverage through intensified investments towards measles elimination is cost-effective but has a high budget impact. Therefore, policy makers should consider market shaping interventions and strategies to mobilize and allocate sufficient resources to increase vaccination coverage and ensure sustained health gains.

## Introduction

Measles is one of the most contagious human diseases, caused by the airborne measles virus. Unvaccinated young children, pregnant women and immunocompromised individuals have the highest risk of complications [1]. Globally, from 2022 to 2023, measles cases increased by 20%, while deaths decreased by 8%, to an estimated 10.3 million cases and over 100,000 deaths in 2023, as per modelled estimates [2]. In Kenya, an estimated 9,000 confirmed cases and 165 deaths (1.8% case fatality) were reported between 2003 and 2016 [3]. More recently, surveillance data indicate approximately 2,900 cases and 18 deaths between January 2024 and February 2025 [4]

Beyond the health impact, measles also imposes a significant economic burden. Societal costs in low-and middle-income countries (LMICs) range from 2018 US$15 in Uganda to US$18 in Bangladesh for outpatient costs and US$60 to US$159 for hospitalized patients in Uganda and Bangladesh respectively [5,6].

World Health Organization (WHO) recommends two doses of measles containing vaccine (MCV) to be administered to all children, as this gives a high level of protection [7]. Given how infectious the disease is, a high coverage rate of 95% is needed to provide herd immunity and prevent its spread [7]. However, global vaccine coverage of the first MCV dose has plateaued over the last decade, remaining at 84% in both 2014 and 2024. While the second dose coverage increased from 59% in 2014 to 76% in 2024 [8]. This stagnation has led to a gap resulting in significant measles outbreaks. For instance, the number of countries that experienced disruptive measles outbreaks increased from 36 to 57 between 2022 and 2023 [2]. Countries including those in the African region have committed to the immunization agenda 2030, with the goal to eliminate measles and achieve MCV1 and MCV2 vaccine coverage of 95% in at least 80% of African countries by 2030 [9,10].

Kenya has been committed to measles control since 2002 through the provision of the first dose of MCV to all infants at 9 months of age, and the introduction of the second dose (MCV2) into the routine immunization in 2013 to children aged 18 months [11]. In addition, periodic supplementary immunization activities (SIAs) have been conducted to provide children with a second opportunity for measles vaccination, alongside case-based surveillance through laboratory confirmation and epidemiological linkage to outbreaks [11].

Despite these efforts, several challenges to measles control persist in Kenya. The country borders conflict-afflicted countries, including South Sudan and Somalia, resulting in substantial cross-border movement of immigrants and refugees, many of whom may be unvaccinated against measles [12]. Kenya has also experienced delays in the implementation of SIAs, which have contributed to measles outbreaks within the country [13,14]. Furthermore, improvements in measles vaccination coverage have been modest, and in some periods, stagnant. For example, between 2018 and 2024, national MCV1 coverage increased from 78% to 82% and MCV2 coverage improved from 42% to 60%.

To achieve the Immunization Agenda 2030 targets for measles elimination, countries will need to make additional investments and intensify efforts to increase vaccination coverage. Generating evidence on the value for money and affordability of vaccine strategies aimed at improving measles vaccination coverage is therefore important for informed vaccine policy making. Therefore, this study aims to conduct a cost-effectiveness and budget impact analysis (BIA) of scaling up vaccination coverage in Kenya, towards the goal of measles elimination.

## Methods

### Ethics statement

Ethical approval for the study was obtained from the Kenya Medical Research Institute Scientific and Ethics Review Unit (KEMRI/SERU/CGMRC-4983).

### Study design and intervention comparators

A cost-effectiveness analysis was conducted to evaluate the costs and health outcomes of alternative strategies to improve measles vaccination coverage in Kenya. The analysis adopted a health system perspective over a 10-year time horizon, with a new birth cohort entering the model each year. Primary outcomes included disability adjusted life years (DALYs), costs borne to the provider, and associated changes in health outcomes. All costs were derived from secondary sources and reported in 2025 US dollars (USD). Costs incurred in other years were adjusted for inflation using GDP deflators. Costs and health outcomes were discounted at 3%, as recommended by the WHO [15].

An incremental cost effectiveness analysis was done comparing three vaccination coverage scenarios, each evaluated against the next best alternative to generate incremental cost-effectiveness ratios (ICERs). Specifically, the constant improvement scenario was compared against the base case, and the intensified improvement scenario against the constant improvement scenario.

The base case (status quo) assumed routine measles vaccination and SIAs continue, but no additional investment is made to improve coverage. Current 2024 national coverage levels for MCV1 and MCV2 were therefore maintained throughout the 10-year time horizon and SIAs were assumed to be conducted every 5 years among children under five years of age, regardless of previous vaccination status consistent with current practice in the country.

Scenario 1 (constant improvement) assumed current funding is maintained to ensure incremental improvements in vaccination coverage based on historical trends in Kenya (10-year trend for MCV1 and 5-year trend for MCV2). As a result, coverage was assumed to increase annually by 0.3% for MCV1 and 2% for MCV2. SIAs were assumed to continue at the same frequency and target population as in the base case.

Scenario 2 (intensified investment) assumed additional investments to reach hard-to-reach populations, resulting in vaccination coverage reaching 95% for both MCV1 and MCV2 by year 10, with SIAs assumed unchanged from the base case. The detailed vaccination strategies are presented in Table 1.

**Table 1:** Intervention comparators within a 10 year time horizon.

	MCV1 coverage (9 months)	MCV 2 coverage (18 months)	SIA
Baseline	Base year coverage maintained (82%)	Base year coverage maintained (60%)	SIA every 5 years (95% coverage, children <5 years, irrespective of vaccination status)
Scenario 1-Constant improvement	0.3% annual increase (till 84.7%)	2% annual increase (till 78%)	SIA every 5 years (95% coverage, children <5 years, irrespective of vaccination status)
Scenario 2 -Intensified investment	Until 95% coverage	Until 95% coverage	SIA every 5 years (95% coverage, children <5 years, irrespective of vaccination status)

### Markov model

A static Markov model was developed in Microsoft excel with annual cycles over the 10-year time horizon. A new birth cohort was introduced each year.

At birth, children are assumed to be protected by maternal antibodies (MI). During the first year of life, this protection wanes and children either receive the first dose of the measles vaccine (V1) or become susceptible to infection (S). In the second year of life, children may receive the second dose of the vaccine (V2). Additional vaccination opportunities occur through national SIAs targeting children under 5 years, irrespective of prior vaccination status.

**Fig 1:**
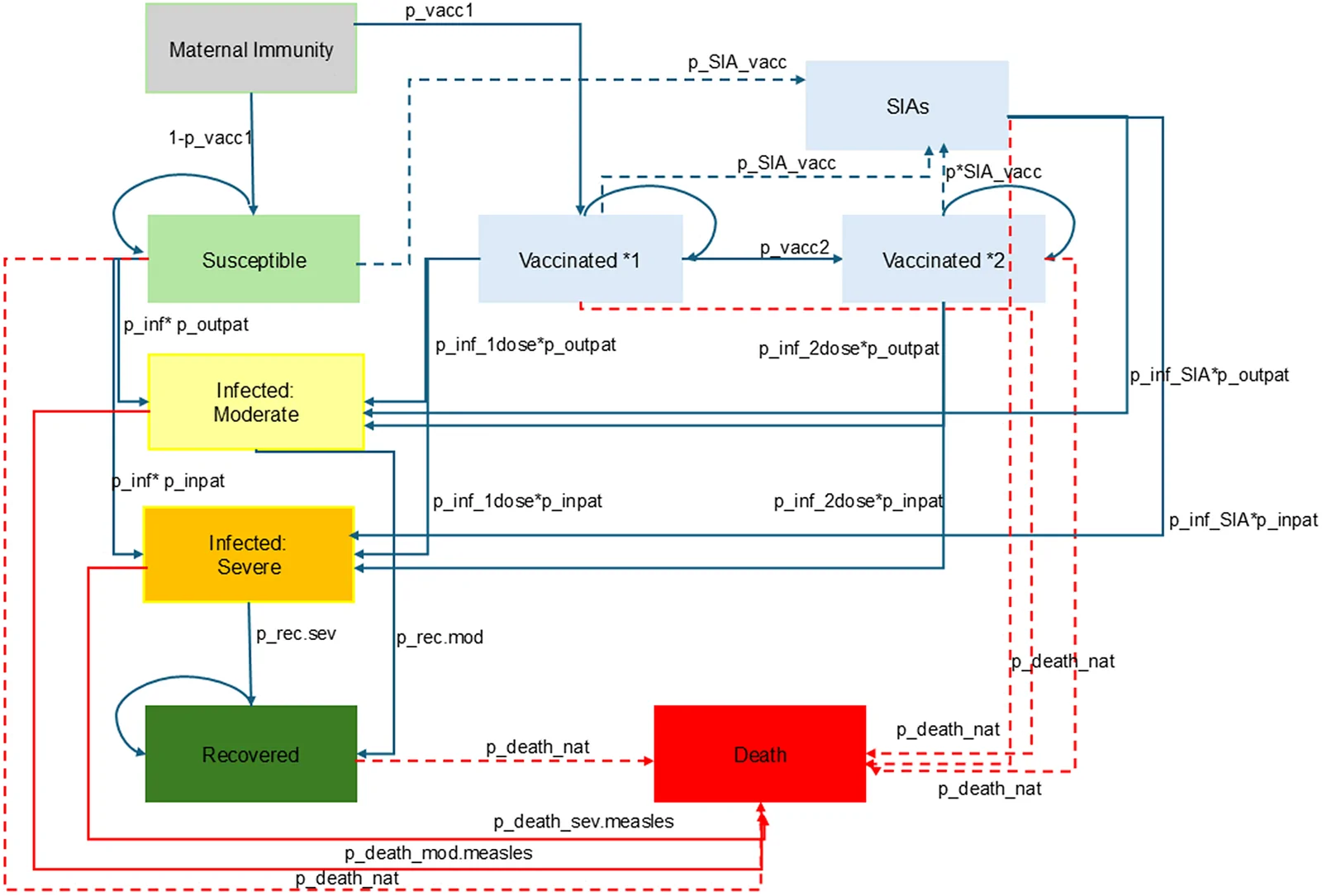
Model Structure.

Both susceptible and vaccinated children may acquire measles infection, with different probabilities reflecting on the vaccine’s effectiveness. An infection was categorized as moderate (IM) and assumed to be managed in an outpatient setting or severe (IS) requiring inpatient care. Following infection, children may recover (R) or die (D) from measles. Age-specific all-cause mortality was also incorporated to account for deaths unrelated to measles. However, the results presented in this paper report only measles attributable deaths and DALYs. Fig 1 is a diagrammatic representation of model.

### Input parameters

Table 2 summarises the model input parameters, data sources and key assumptions. The input parameters are described in detail in subsequent sub-sections.

**Table 2:** Markov model input parameters.

Variable	Estimate	Description/Notes	Data source
**Vaccine coverage estimates**			
MCV1 coverage (base case)	81.6%	Current national coverage	MOH data [16]
MCV2 coverage (base case)	59.2%	Current national coverage	MOH data [16]
Target MCV1 coverage -Scenario 1	82.8% -(first 5years) 84.3% (last 5 years)		Based on historic trends
Target MCV2 coverage -Scenario 1	67.7% -(first 5years) 77.7% (last 5 years)		Based on historic trends
Target MCV1 coverage -Scenario 2	90% -(first 5years) 95% (last 5 years)		Assumption
Target MCV2 coverage -Scenario 2	75% -(first 5years) 95% (last 5 years)		Assumption
Target SIA coverage	95% coverage every 5 years		Assumption
**Epidemiological estimates**			
Proportion of reported measles cases and deaths	10%		[17]
Probability of a susceptible (unvaccinated) child gets infected with measles (p_inf)	1.74%	An average of 700 measles cases per year [18] was assumed, with 55% occurring in children under 5 years [3]. After adjusting for underreporting, these cases were assumed to occur among unvaccinated children in the birth cohort and were used to estimate the annual probability of infection among those susceptible.	[3,18]
Vaccine efficacy (VE)	MCV1: 84% (IQR: 72–95%) MCV2: 94% (IQR: 88%-98.3%)		[19]
Probability of infection after MCV1 (p_inf_1dose)	0.279%	(1-VE after dose1)*p_inf	
Probability of infection after MCV2 (p_inf_2dose)	0.103%	(1-VE after dose2)*p_inf	
Probability of infection after SIA (p_inf_SIA)	0.19%	Average of protection from MCV1 and MCV2	Assumption
Proportion of inpatients (severe measles cases)	8% (5-10%)	Unreported cases: 5% severe, 95% outpatient. Reported cases: 36% hospitalization	Assumption and [3]
Proportion of outpatient (moderate measles cases)	92% (90-95%)		
Probability of deaths from moderate measles (case fatality)	1.92% (95% CI 1.86%-1.97%)	Case fatality rates for community based and hospitalized measles in sub-Saharan Africa	[20]
Probability of deaths from severe measles (case fatality)	7.67% (95% CI 7.31% – 8.07%)		
Probability of deaths from natural causes	4%	Under 5 mortality (41 per 1000)	[21]
Probability of recovering from moderate 4measles	98.08%	1-Case fatality for moderate measles	
Probability of recovering from severe measles	92.33%	1-Case fatality for moderate measles	
**Cost estimates (2025 US$)**			
Cost of vaccine procurement	1.28	Includes vaccine, and injection device costs and their wastage rates	[22]
Cost of routine vaccine delivery (base case)	2.68	Based on MCV 1 coverage (costs for 80–89% coverage used)	[23]
Cost of continued routine delivery (scenario 1)	2.68	Based on MCV 1 coverage (costs for 80–89% coverage used)	
Cost of intensified routine delivery (scenario 2)	3.32	Based on MCV 1 coverage (costs for 90% + coverage used)	
Cost of campaign delivery	0.91		
Cost of outpatient treatment	4.32	Included consultation costs, laboratory tests, medications, staff costs, utilities and building space.	[24]
Cost of inpatient treatment	159.40	Included hospital bed days, staff time, laboratory tests, medications, and other supportive care costs (e.g., blood transfusions, oxygen supplementation, phototherapy and nutrition items)	[25]
**DALY parameters**			
Disability weight for moderate measles	0.051 (0.032 – 0.074)		[26]
Disability weight for severe measles	0.133 (0.088 – 0.19)		
Duration of measles disease	8.5 days (7–10 days)		Assumption
Average age of death	2.57	Weighted average of 0–5years population used	Assumption
Life expectancy	63.66		[27]

i) Demographics and Vaccine Coverage Estimates

The estimated 2019 birth cohort of 1,105,074 children was projected to 2024 using an annual population growth rate of 2.2.% [28]. The resulting 2024 birth cohort was then further projected over the 10-year time horizon, applying the same annual growth rate to account for demographic changes.

Based on 2024 national coverage estimates, the probability of a susceptible child receiving MCV1 was 81.6%, while the probability of receiving MCV2 was 59.7% in the base case scenario [16].

Under the constant improvement scenario, MCV1 and MCV2 coverage was assumed to be 82.8% and 67.7% for the first five years and 84.3% and 77.7% and for the final five years of the analysis, respectively. In the intensified investment scenario, MCV 1 and MCV2 target coverage was assumed to be 90% and 75% respectively in the first five years and 95% for both doses in the final five years.

For SIAs, assuming 95% coverage would be achieved over a five-year period, an annual probability of vaccination of 19% was applied.

ii) Epidemiological Estimates

Epidemiological parameters were used to estimate measles infection risk and disease severity among children. Reported measles cases and deaths were adjusted for under-reporting, with the assumption that only 10% of cases are reported. This assumption is consistent with evidence suggesting that only 2–10% of true cases of measles (under-reporting may be as high as 10–50 times higher) are reported in sub-Saharan Africa [17]. Among unreported cases, majority (95%) were assumed to be outpatient and 5% required hospitalization, while among reported cases, 36% were assumed to require hospitalization [3]. Overall, this resulted in the assumption that 8% and 92% of all measles cases required hospitalization and outpatient services respectively.

The annual probability of infection among susceptible (unvaccinated) children in the birth cohort was calculated based on an average of 700 reported cases per year in Kenya over 2014–2025, since measles cases differ substantially between outbreak and non-outbreak years [18], of which 55% occurred in children under 5 years [3]. This estimate was further adjusted for under-reporting. Vaccine effectiveness was assumed to be 84% (IQR 72–95%) after MCV1 and 94% (IQR: 88.3-98.3%) after MCV2 [19], which were used to calculate post-vaccination infection probabilities. Protection following SIAs was assumed to reflect the average effectiveness of MCV1 and MCV2.

The case fatality rates for measles were assumed to be 1.92% (95% CI 1.86%-1.97%) for moderate and 7.67% (95 CI 7.31% – 8.07%) for severe cases [20]. An under 5 mortality rate of 41 per 1,000 live births was used as the probability of death from natural causes [21]

iii) Cost estimates

Vaccination costs included vaccine procurement and delivery costs. Vaccine procurement costs comprised the costs of the vaccine and injection devices, which included injection syringes, reconstitution syringes and safety boxes. Wastage rates were accounted for: 5% for the syringes and 32% for the vaccine [22].

Vaccine delivery costs were estimated based on the MCV1 coverage levels and were sourced from Levin et al (2023), who modelled the elasticity of measles vaccine delivery costs to coverage in low-and middle-income countries [23]. As reported in literature, delivery costs increased with increased coverage (non-linear), attributed to an increase in activities related to communication and social mobilization, cold chain expansion, and surveillance. These activities were assumed to be intensified, in order to access the hard-to-reach populations [23].

Outpatient treatment costs were estimated at US$4.20 as reported by a primary health cost analysis done by the Kenyan Ministry of Health [24]. These outpatient costs included direct costs to the provider such as consultation costs, laboratory tests, medications, staff costs, utilities and building space [24]. Inpatient treatment costs were estimated at a mean of USD 155.06, derived from a Kenyan cost analysis of measles hospitalizations among children [25]. These costs included direct costs to the provider including hospital bed days, staff time, laboratory tests, medications, and other supportive care costs (e.g., blood transfusions, oxygen supplementation, phototherapy and nutrition items) [25].

iv) Disability-adjusted life years

The outcome of the cost-effectiveness analysis was reported in terms of disability adjusted life years (DALYs); the sum of years of life lost (YLL) and years lost due to disability (YLD) [29].

YLL is estimated as [29]:


$$\begin{array}{c} \text{YLL}=\frac{{KCe}^{ra}}{(r+\beta {)}^{\text{2}}}\left\{e^{-(r+\beta )(L+a)}\left[-(r+\beta )(L+a)-\text{1}\right]-e^{-(r+\beta )a}\left[-(r+\beta )a-\text{1}\right]\right\}+\frac{\text{1}-K}{r}(\text{1}-e^{-rL}) \end{array}$$

*Where*: K = age weighting modulating factor; C = adjustment constant for age weights; r = discount rate; a = age of death; β = parameter from the age weighting function; L = standard life expectancy at age of death

YLD is estimates as [29]:


$$\begin{array}{c} \text{YLDs}=\text{D}\{\frac{{KCe}^{ra}}{(r+\beta {)}^{\text{2}}}\left\{e^{-(r+\beta )(L+a)}\left[-(r+\beta )(L+a)-\text{1}\right]-e^{-(r+\beta )a}\left[-(r+\beta )a-\text{1}\right]\right\}+\frac{\text{1}-K}{r}\left(\text{1}-e^{-rL}\right)\} \end{array}$$

*Where*: K = age weighting modulating factor; C = adjustment constant for age weights; r = discount rate; a = age of onset of disability; β = parameter from the age weighting function; L = duration of disability; D = disability weight

DALYs were calculated considering no age-weighting, a discount rate of 3% and the Kenyan 2024 standard life expectancy [27]. An average age of death of 2.57 years among children under five was assumed, based on the weighted average age of 0–5 population. The duration of illness was assumed to be 8.5 days (range 7–10 days), and disability weights were applied. Disability weights for moderate measles was 0.051 (0.032 – 0.074) and for severe measles 0.133 (0,088 – 0.19) [26].

### Cost-effectiveness analysis

The ICER was the cost-effectiveness measure calculated as the net change in total costs and DALYs averted between comparators. The ICER was compared against an opportunity cost threshold for Kenya, as estimated by Ochalek [30,31]. In line with this approach, 0.5 times the GDP per capita (2025 USD 1,280) was used as the threshold [32].


$$\begin{array}{c} ICER=\frac{{Cost}_{index}-\;{Cost}_{baseline}}{{DALY}_{baseline}-{DALY}_{index}} \end{array}$$

*Where*: Cost_index_ = cost of strategy of interest; Cost_baseline_ = cost of next less effective strategy; DALY_index_ = total DALYs under the strategy of interest; DALY_baseline_ = total DALYs under the next less effective strategy

### Sensitivity analysis

A univariate sensitivity analysis was done to determine the robustness of the cost-effective results to key parameter uncertainties, including vaccine procurement costs, vaccine delivery costs and the reporting rate. Vaccine procurement costs were doubled, to reflect potential future increases in vaccine prices as Kenya transitions from GAVI support. Vaccine delivery costs were varied by 30% above and below the base cost. The reporting rate was varied from 2% to 20% to account for uncertainty in case detection.

The primary analysis assumed direct vaccine protection and a standard infection probability among susceptible (unvaccinated) individuals, based on current measles transmission and vaccination coverage in Kenya. Given that the Intensified investment scenario aims to achieve 95% coverage for both MCV1 and MCV2, consistent with WHO recommendations for measles elimination and achievement of herd immunity, an additional scenario analysis explored the potential indirect protection associated with increased population immunity. The infection probability among the unvaccinated individuals was reduced by 25%, 50%, 75% and 82% for the intensified investment scenario. The 82% reduction was informed by Amela Heras et al, who reported a corresponding reduction in measles cases among unvaccinated individuals associated with a vaccination programme [33]. These reductions were exploratory and intended to assess the potential impact of indirect protection.

A probabilistic sensitivity analysis was also performed to evaluate the joint uncertainty in model parameters and their influence on the ICERs. Parameter uncertainty was informed by reported confidence intervals where available and for costs, a range of 20% increase or decrease around the base-value was assumed. Gamma distributions were used for cost parameters while Beta distributions were s used for parameters bounded between 0 and 1.

### Budget impact analysis

A BIA was conducted to assess the affordability of the alternative vaccination strategies compared with the base scenario. The BIA analysis was done over a time frame of 5 years to inform short-to-medium term financial planning.

The BIA included the total costs of treatment for the consecutive birth cohorts and the total vaccination costs (procurement + delivery costs) over the time horizon. The net budget impact was estimated as the difference in total health system costs (treatment cost + vaccination costs) between each vaccination scenario and the base case, representing the additional fiscal impact to the health system.

To assess affordability, a standardized budget impact threshold (BIT) was calculated, expressed as the incremental per capita annual health expenditure per 1 million inhabitants (and as a proportion of current health expenditure) [34].


$$\begin{array}{c} {BIT}_{std}=\frac{{BIT}_{Net}}{{Population}_{tot\;}X\;{HE}_{pc}}\;X\;\text{1}\;\text{0}\text{0}\text{0}\;\text{0}\text{0}\text{0} \end{array}$$

*Where*: BIT_standard_ = standardised BIT, expressed as the incremental per capita annual health expenditure per 1 million inhabitants; Population_tot_ = number of people covered by the health system; HE_pc_ = annual per capita health expenditure

The 2019 population was projected forward using an annual growth rate of 2.2%, resulting in an estimated population of 53,031,700 for Year 1 (representing 2024) [28]. The same growth rate was employed for the subsequent years. The 2023 annual per capita health expenditure of USD 84.96 [35] was used for year 1, and an inflated by 5% in the following years, reflecting the local inflation rate, under the assumption of a fixed exchange rate over the projection period.

The standardized BIT was classified based on the incremental per capita annual health expenditure per 1 million inhabitants (and as a proportion of current health expenditure) as follows [34]: low < 50 (<0.005%), moderate: 50 to <100 (0.005% to <0.01%), high: 100 to <200 (0.01% to <0.02%) and very high: 200 or more (>=0.02%).

### Patient and public involvement

No patients were involved in this study. However, policy makers were involved in the development of the research questions, and the study results will be disseminated to key policy makers.

## Results

### Clinical outcomes and cost-effectiveness analysis

The health and economic outcomes over a 10-year time horizon, and for 10 consecutive birth cohorts are reported in Table 3. The constant improvement scenario resulted in 5.9% (11,317 (8,761 – 14,396)) fewer cases and 5.7% (213 (161–276)) fewer deaths compared to the base scenario. An increase in vaccine coverage in the intensified investment scenario would result in a further 25% (45,370 [33] (33,263 – 65,576)) reduction in cases and a further 879 (626-1,284) reduction in deaths compared to the constant improvement scenario.

**Table 3:** Clinical outcomes, costs and cost-effectiveness of different vaccination strategies over a 10-year horizon from a health system’s perspective.

	Averted cases- Median (2.5-97.5th percentile)	Averted deaths- Median (2.5-97.5th percentile)	Total costs (million USD) Median (2.5-97.5th percentile)	Total DALYs Median (2.5-97.5th percentile)	Mean ICER
Base scenario	–	–	80.64 (61.15 – 104.64)	105,969 (83,158 – 139,488)	
Scenario 1 (Constant improvement)	11,317 (8,761 – 14,396)	213 (161 -276)	88.55 (67.12 – 114.23)	99,924 (77,841 – 132,392)	1,269 (1,205– 1,333)
Scenario 2 (Intensified investment)	45,370 (33,263 – 65,576)	879 (626 – 1,284)	115.83 (86.60 – 151.85)	74,730 (56,741 – 102,709)	1,093 (1,029 – 1,157)

As vaccine coverage increased the total costs rose, from USD 80.64 (61.15 – 104.64) million in the base scenario to USD 115.83 (86.60 – 151.85) million in the intensified investment scenario, while total DALYs decreased with higher vaccine coverage.

Under scenario 1, the constant improvement strategy was cost-effective (ICER = USD 1,269 (1,205 - 1,333) compared to the base scenario. However, further increasing vaccination coverage in the intensified investment scenario was more cost-effective (ICER = USD 1,093 (1,029 - 1,157) compared to scenario 1.

### Sensitivity analysis

**Fig 2:**
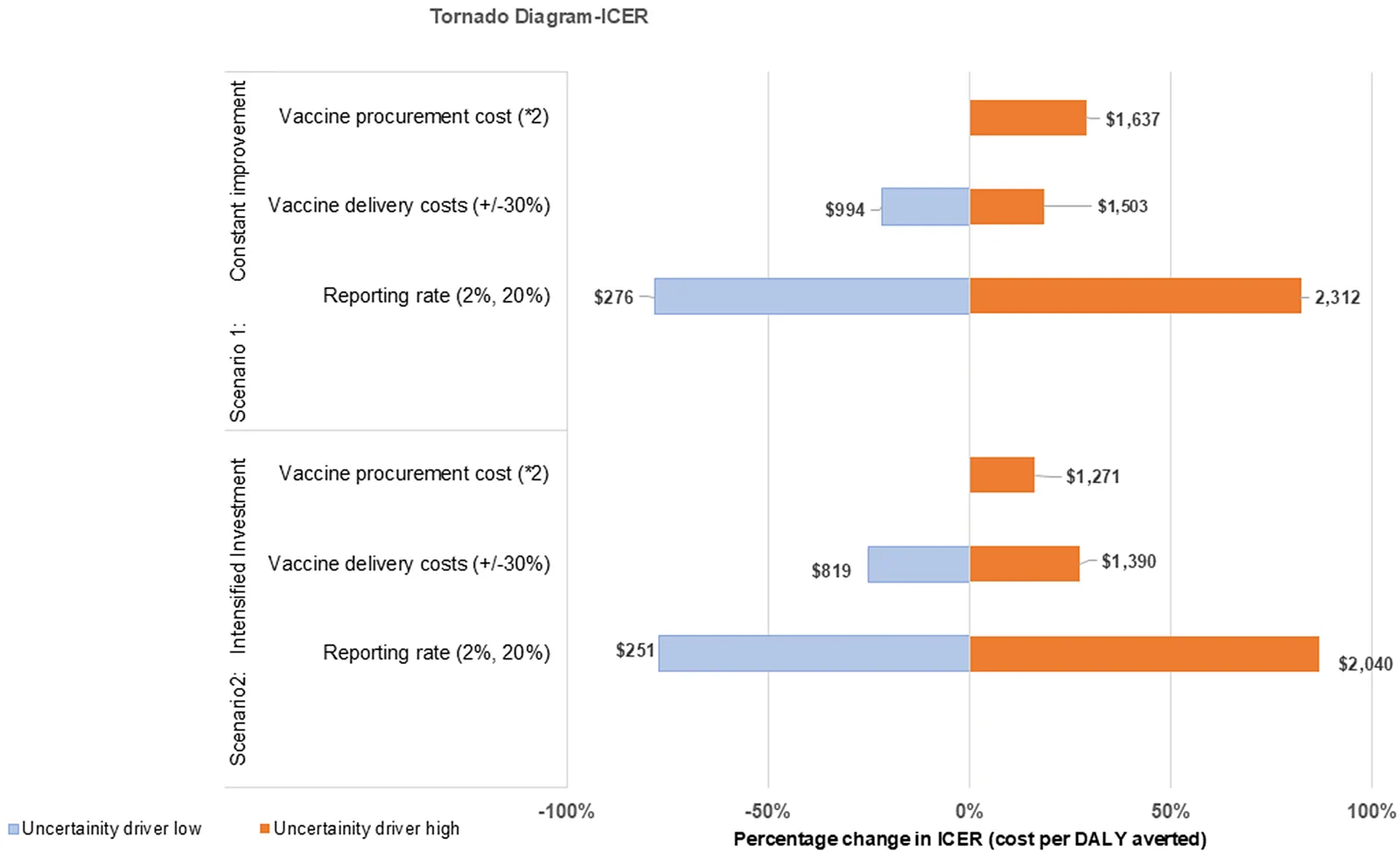
One-way sensitivity analysis of vaccine prices and reporting rate on ICERs.

Fig 2 presents the effects of vaccine prices, and the reporting rate on the ICER. The reporting rate had the largest effect on the ICER: leading to a 77–78% decrease and 82%-87% increase in ICERs across the different vaccination scenarios. Vaccine procurement costs led to 16% and 29% increase in the ICER in Scenario 1 and 2 respectively.

**Fig 3:**
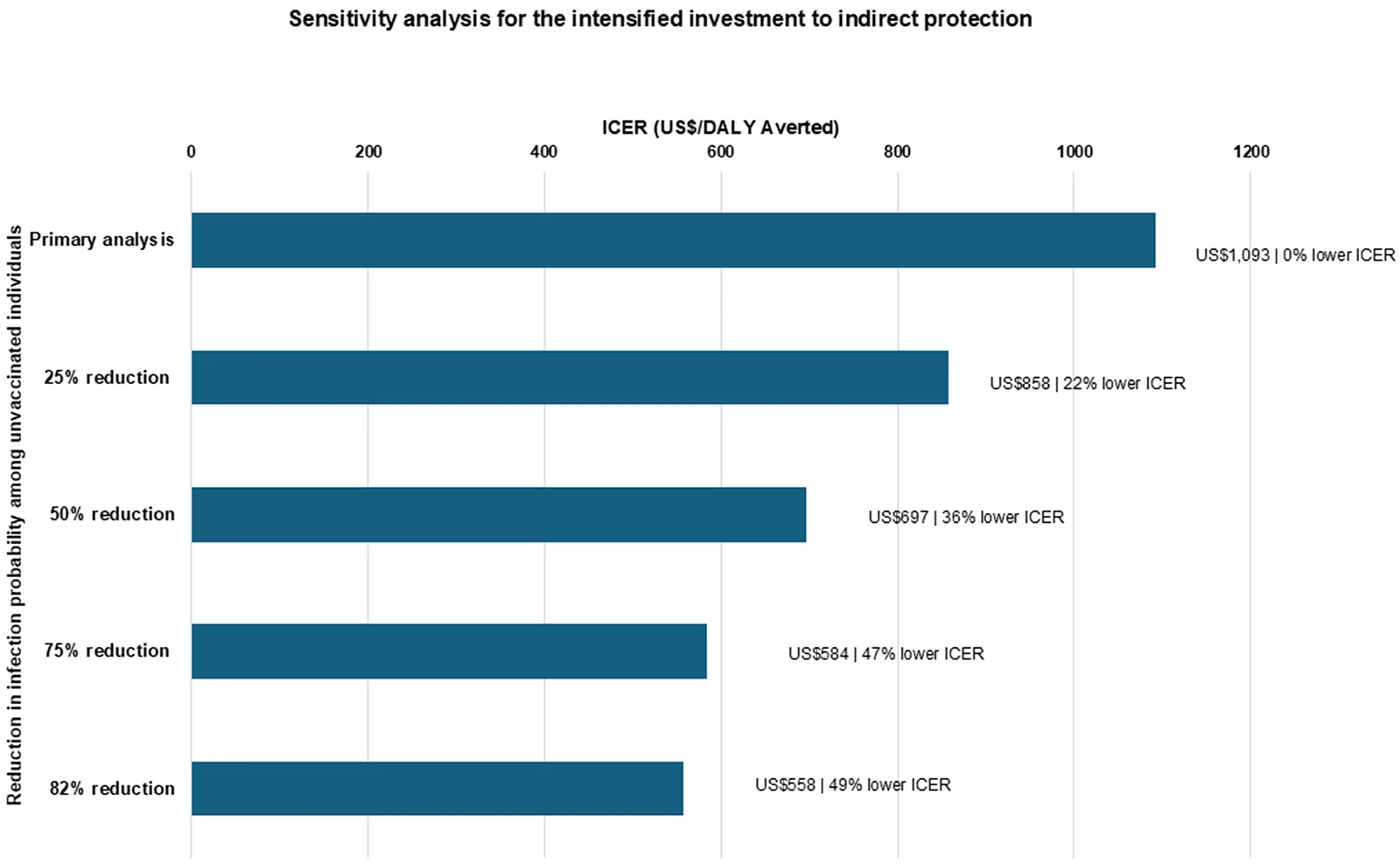
Sensitivity analysis for the intensified investment to indirect protection.

The sensitivity analysis assessing the potential effect of indirect protection resulting from higher vaccine coverage and population immunity is presented in Fig 3. Reducing the infection probability among unvaccinated individuals in the intensified investment scenario by 25% and 82% resulted in a 22% (USD 858 per DALY averted) and 49% (USD 558 per DALY averted) lower ICERs, respectively, compared with the primary analysis.

**Fig 4:**
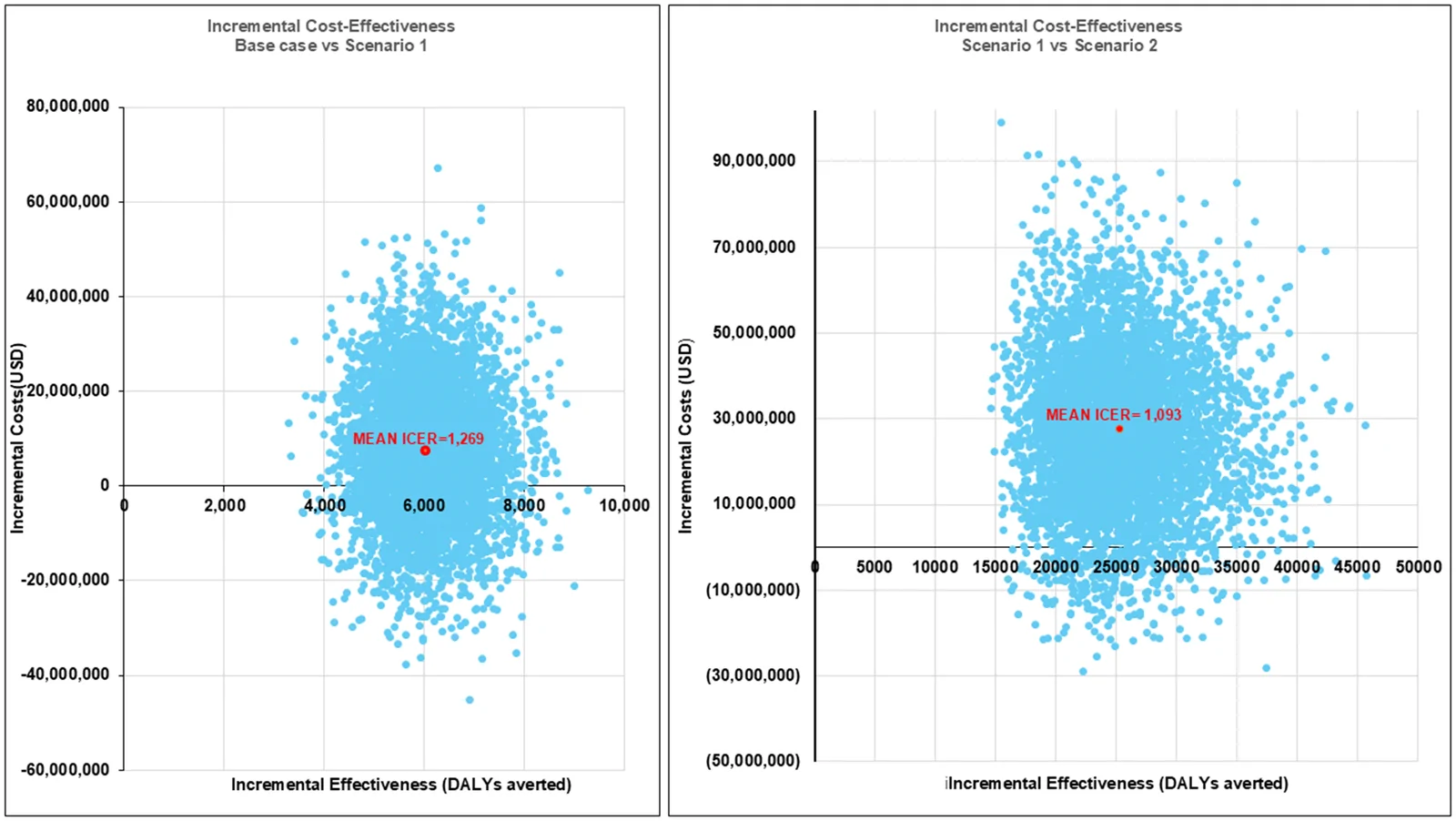
Probabilistic sensitivity analysis of different vaccination strategies.

The findings of the probabilistic sensitivity analysis from a health-system’s perspective are shown in Fig 4. For scenario 1, 71% of the cost-effect pairs were in the north-east quadrant (implying the intervention is more costly but also more effective) and 29% fell in the south-east quadrant (implying less costly and more effective) compared to the base case. For scenario 2, 94% of the replications were in the north-east quadrant and 6% were in the south-east quadrant relative to scenario 1.

**Fig 5:**
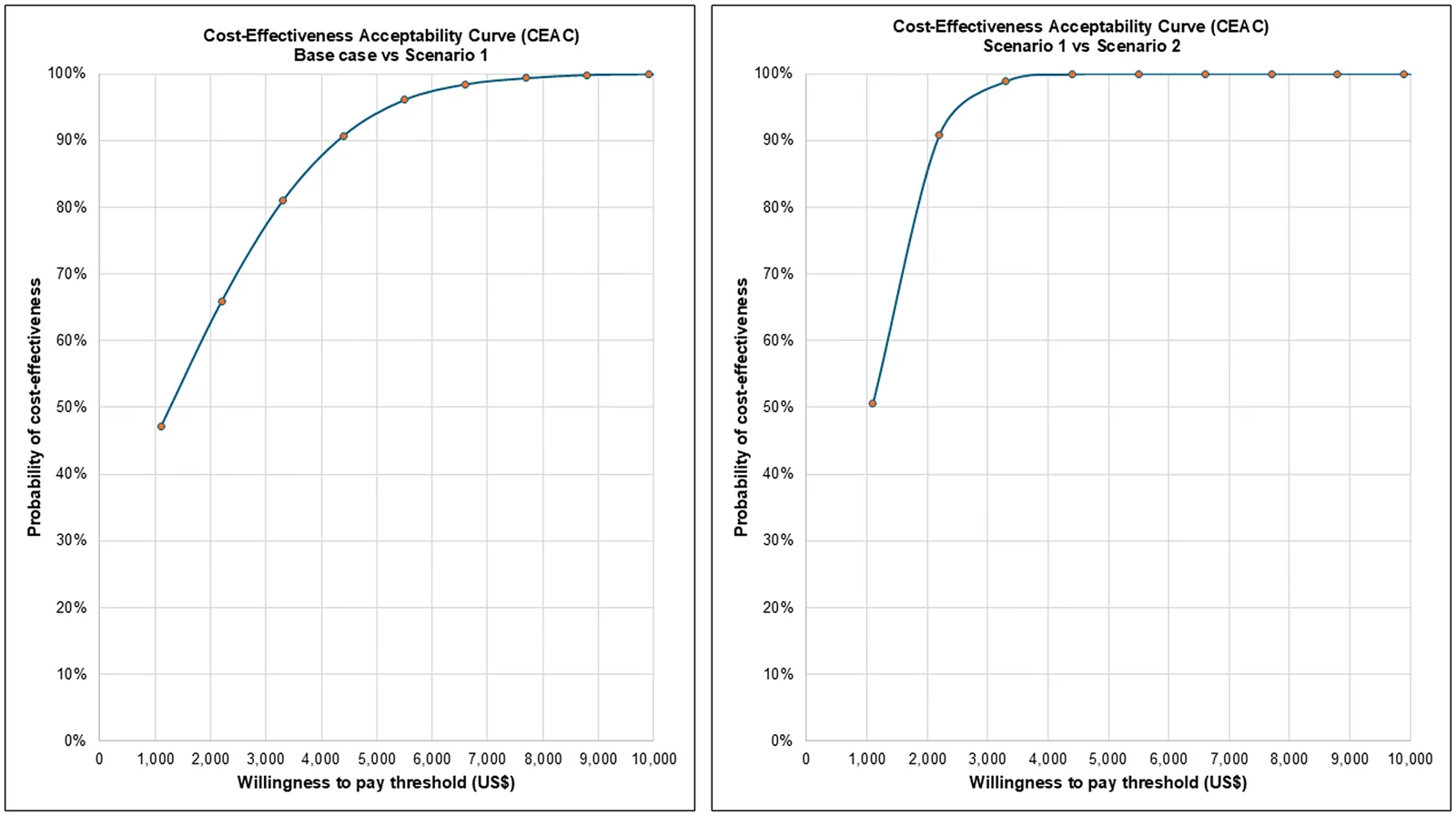
Cost-effectiveness acceptability curves (CEAC) over a range of cost-effectiveness thresholds.

Fig 5 presents the cost-effectiveness acceptability curves (CEAC) based on a range of cost-effectiveness thresholds. For the constant improvement scenario compared to the base scenario, there is a 50% and 100% probability that it would be cost-effective given a USD 1,100 and USD 8,800 willingness to pay threshold respectively. For the intensified investment scenario, there is a 100% probability that it would be cost-effective at a USD 4,400 willingness to pay threshold compared to the constant improvement scenario.

### Budget impact analysis

The net budget impact of the constant improvement scenario increased from USD 59,962 in Year 1 to USD 471,149 in Year 5 compared to the base case. This resulted in a total budget impact of USD 1.88 million over the 5 years. A similar trend was observed with the intensified investment scenario compared to baseline, where the net budget impact increased from USD 1.13 million in year 1 to USD 2.75 million in year 5, totalling to USD 11.92 across the five-year time horizon. This is reported in Table 4

**Table 4:** Budget impact analysis for the different measles vaccination coverage strategies (USD).

	Year 1	Year 2	Year 3	Year 4	Year 5	Total
**Base case**						
Treatment costs	–	116,548	196,894	264,365	324,522	902,329
Vaccination costs	4,076,906	7,125,084	7,609,721	7,831,643	8,044,906	34,688,260
Total Health System Costs	4,076,906	7,241,632	7,806,615	8,096,008	8,369,428	35,590,589
**Scenario 1: Constant improvement**						
Treatment costs	–	112,814	187,537	251,822	309,816	861,989
Vaccination costs	4,136,868	7,553,117	8,080,916	8,309,652	8,530,761	36,611,314
Total Health System Costs	4,136,868	7,665,931	8,268,453	8,561,474	8,840,577	37,473,303
^**+**^ **Net Budget Impact**	**59,962**	**424,299**	**461,838**	**465,466**	**471,149**	**1,882,714**
**Scenario 2: Intensified Investment**						
Treatment costs	–	90,381	143,531	192,463	238,527	664,902
Vaccination costs	5,211,413	9,758,789	10,366,688	10,624,364	10,880,352	46,841,606
Total Health System Costs	5,211,413	9,849,170	10,510,219	10,816,827	11,118,879	47,506,508
^**+**^ **Net Budget Impact**	**1,134,507**	**2,607,538**	**2,703,604**	**2,720,819**	**2,749,451**	**11,915,919**

+The Net Budget Impact subtracts the total health system cost (treatment +vaccination costs) of each scenario to the base case scenario

The standardized budget impact thresholds are reported in Table 5. The standardized budget impact for the constant improvement scenario was low in year 1 (0.0013%) but moderate in years 2–5 (0.0079% to 0.0089%). This implies that, compared to the base case, Scenario 1 would generate a budget impact equivalent to providing health coverage to 13 additional individuals per one million population in Year 1 and between 79–89 additional individuals per one million population in years 2–5. Based on Kenya’s total population, this corresponds to approximately 706 individuals in year 1 and between 4,562–4,931 individuals annually in years 2–5.

**Table 5:** Standardized budget impact threshold by vaccination strategy.

		Year 1	Year 2	Year 3	Year 4	Year 5
**Health Expenditure (per capita)**		84.96	89.21	93.67	98.35	103.27
**Population**		53,031,700	54,198,398	55,390,763	56,609,359	57,854,765
**Standardised budget impact threshold**	**Scenario1: Constant improvement**	13 (0.0013%) (Low)	88 (0.0088%) (Moderate)	89(0.0089%) (Moderate)	84 (0.0084%) (Moderate)	79 (0.0079%) (Moderate)
	**Scenario 2: Intensified investment**	252(0.0252%) (Very High)	539(0.0539%) (Very High)	521(0.0521%) (Very High)	489(0.0489%) (Very High)	460(0.046%) (Very High)

The intensified investment scenario had a very high standardized budget impact over the 5-year period, increasing from 0.0252% in Year 1 to between 0.046% to 0.0539% in years 2–5. This corresponds to a budget impact equivalent to providing health coverage to 252 additional individuals per one million population in Year 1 and 460–539 additional individuals per one million in years 2–5. This is equivalent to approximately 13,353 individuals in year 1 and between 26,624–29,230 people in year 2–5, when scaled to Kenya’s total population.

## Discussion

In this analysis we assessed the cost-effectiveness and budget impact of scaling up vaccination coverage in Kenya. Our findings show that both vaccination scale-up scenarios are cost-effective. However, the intensified investment strategy, that ramps up coverage towards the 95% threshold required for measles elimination strategy, was more cost-effective and resulted in substantial reduction in measles cases and deaths.

These findings support prioritization of measles vaccination scale up in Kenya, especially in the context of stagnating vaccination coverage and recurrent measles outbreaks [8,12–14]. Achieving and sustaining high coverage will require targeted investments in routine immunization systems such as cold chain infrastructure, social mobilization, surveillance and community engagement efforts to address vaccine hesitancy. Importantly, these investments aimed at strengthening measles control are unlikely to operate in isolation. Improvement in the supply chain infrastructure and communication, among other investments, may generate a positive spillover effect for improving coverage for other antigens within the expanded programme on immunization and potentially for broader primary health care services. Although these system-wide benefits are not quantified in our analysis, they may further enhance the overall value proposition of intensified investment.

Our findings are consistent with prior modelling work done in low-income and middle-income settings. For example, analysis by Levin et al., used a dynamic transition model to assess different scenarios of ramping up the measles rubella vaccination [23]. Their findings reported that strategies that accelerate vaccination coverage above current trends were generally more cost-effective, with faster ramp-up scenarios being the most cost-effective [23]. Other modelling evidence further suggests that achieving measles elimination requires not only high national coverage levels but also equitable distribution of coverage to interrupt endemic transmission [36]. In our study, national level vaccination coverage estimates were used, which may mask subnational disparities. However, measles outbreaks often disproportionately affect the underserved and hard-to-reach populations where immunity gaps persist. This highlights the need for policy makers to also integrate equity considerations when scaling-up vaccination coverage interventions, with a focus on low-coverage counties and marginalized communities in Kenya.

While our results reinforce, global elimination recommendations by showing that the intensified investment is more cost-effective, affordability remains a key challenge. The budget impact associated with rapidly scaling up coverage to elimination levels is substantial when assessed against Kenya’s overall current health expenditure. This highlights that although it is cost-effective, there may be implementation barriers if the fiscal space is constrained. Policy makers may therefore need to consider efficiency gains within the immunization programs and market-shaping strategies to procure the vaccine at lower prices. In addition, innovative and sustainable domestic resource mobilization strategies will be essential to ensure long-term financial sustainability, especially in the context of decreasing external funding and transitions from GAVI support. Some limitations should be considered when interpreting these findings. The static Markov model does not capture measles disease dynamic transmission effects and herd immunity which may underestimate the broader population-level benefits of achieving high coverage. As a result, the findings of this study should be interpreted as conservative estimates of the potential health and economic benefits, as the model may underestimate the overall vaccine benefits. Additionally, we used average annual infection risks and could not model probabilities of outbreaks during the time frame. Second, although there were uncertainties and assumptions around some of the parameters, the sensitivity analyses suggest that the conclusions remain robust. Our study results were highly sensitive to the measles-case reporting rate. This highlights the need for improving surveillance, and the completeness and accuracy of reported measles cases, which would strengthen measles burden estimates, elimination tracking and future economic evaluations. Third, for the budget impact analysis, the costs incurred were focused on the included cohort in the Markov model. As a result, the treatment costs may be underestimated as they only cover the costs for the consecutive birth cohorts included in the model, these excludes the measles treatment costs for the rest of the population. Nonetheless, measles cases are largely reported among the included population. Vaccination costs for SIAs may also be underestimated in the budget impact analysis as in practice, it is given to all children under 5 years.

Future research could consider incorporating dynamic transmission modelling to better capture the disease dynamic better, model herd immunity as well as conducting subnational analyses to identify priority areas for targeted inventions and quantifying health system spillover benefits.

## Conclusion

Our findings suggest that accelerating measles coverage through intensified investments towards measles elimination is cost-effective in Kenya but has a high budget impact. Therefore, policy makers should consider market shaping interventions and strategies to mobilize and allocate sufficient resources to increase vaccination coverage and ensure sustained health gains.

## Supporting information

S1 Checklist CHEERS Checklist. (DOCX)
