# Supplementary material for: Cost-effectiveness and budget impact analyses of strategies to improve measles vaccination coverage in Kenya
Source: PLOS Glob Public Health. 2026 Sep 22;6(9):e0006440. doi: 10.1371/journal.pgph.0006440 (PMC13596817; doi:10.1371/journal.pgph.0006440)
Supplement: Supplementary file 2 [file pgph.0006440.s002.docx]

## Cost-Effectiveness and Budget Impact Analyses of Strategies to Improve Measles Vaccination Coverage in Kenya

Response to reviewer comments

Dear Reviewers

Thank you very much for the helpful comments on our paper. In the report below, we have responded to your input within a table that outlines your comment and summarizes our response.

| **Journal Requirements:** |  |
| --- | --- |
| 1. Please note that PLOS Global Public Health has specific guidelines on code sharing for submissions in which author-generated code underpins the findings in the manuscript. In these cases, all author-generated code must be made available without restrictions upon publication of the work. Please review our guidelines at https://journals.plos.org/globalpublichealth/s/materials-and-software-sharing#loc-sharing-code and ensure that your code is shared in a way that follows best practice and facilitates reproducibility and reuse | We thank the editor for highlighting the guidelines on code sharing. Our analysis was conducted using an Excel-based Markov Model. This Markov Model including the formulas and calculations constitutes the analytical “code” for this study. In line with the journal’s policy, we have now made this model publicly available at figshare and have revised the data availability section to read:  “All data underlying the findings are available within the manuscript, In addition, the Excel-Based Markov Model has been deposited in a public repository and is accessible at: https://doi.org/10.6084/m9.figshare.33164015” |
| 2. Please amend your detailed Financial Disclosure statement. This is published with the article. It must therefore be completed in full sentences and contain the exact wording you wish to be published.  i. Please clarify all sources of financial support for your study. List the grants, grant numbers, and organizations that funded your study, including funding received from your institution. Please note that suppliers of material support, including research materials, should be recognized in the Acknowledgements section rather than in the Financial Disclosure.  ii. State the initials, alongside each funding source, of each author to receive each grant. For example: "This work was supported by the National Institutes of Health (####### to AM; ###### to CJ) and the National Science Foundation (###### to AM)." iii. State what role the funders took in the study. If the funders had no role in your study, please state: “The funders had no role in study design, data collection and analysis, decision to publish, or preparation of the manuscript.” iv. If any authors received a salary from any of your funders, please state which authors and which funders. | The financial disclosure statement has now been amended and reads as follows:  “This work was supported by funding from the Wellcome Trust, grant number 228187 (SO, PM, AB, CA, FM, OO, and EB). The funders had no role in the study design, data collection and analysis, decision to publish, or preparation of the manuscript.” |
| 3. We note that your Data Availability Statement is currently as follows: All the data relevant to this study are included in the article.    Please confirm at this time whether or not your submission contains all raw data required to replicate the results of your study. Authors must share the “minimal data set” for their submission. PLOS defines the minimal data set to consist of the data required to replicate all study findings reported in the article, as well as related metadata and methods (https://journals.plos.org/plosone/s/data-availability#loc-minimal-data-set-definition).    For example, authors should submit the following data:    - The values behind the means, standard deviations and other measures reported;  - The values used to build graphs;  - The points extracted from images for analysis.    Authors do not need to submit their entire data set if only a portion of the data was used in the reported study.    If your submission does not contain these data, please either upload them as Supporting Information files or deposit them to a stable, public repository and provide us with the relevant URLs, DOIs, or accession numbers. For a list of recommended repositories, please see https://journals.plos.org/plosone/s/recommended-repositories.    If there are ethical or legal restrictions on sharing a de-identified data set, please explain them in detail (e.g., data contain potentially sensitive information, data are owned by a third-party organization, etc.) and who has imposed them (e.g., an ethics committee). Please also provide contact information for a data access committee, ethics committee, or other institutional body to which data requests may be sent. If data are owned by a third party, please indicate how others may request data access. | We have included all the data relevant in the study within the article. Our analysis was conducted using an Excel-based Markov Model. This Markov Model includes all the data, formulas and calculations used in this study. In line with the journal’s policy, we have now made this model publicly available at figshare and have revised the data availability section to read:  “All data underlying the findings are available within the manuscript, In addition, the Excel-Based Markov Model has been deposited in a public repository and is accessible at: https://doi.org/10.6084/m9.figshare.33164015” |
| 4. Please ensure that your Ethics Statement is available in its entirety at the beginning of your Methods section, under a subheading 'Ethics Statement'. | The ethics statement has been moved to the beginning of the Methods section and reads as follows:  Ethics statement  Ethical approval for the study was obtained from the Kenya Medical Research Institute Scientific and Ethics Review Unit (KEMRI/SERU/CGMRC-4983). |
| 5. We have noticed that you have uploaded Supporting Information files, but you have not included a list of legends. Please add a full list of legends for your Supporting Information files after the references list. | This has now been included after the reference list |
| 6. Please provide separate figure files in .tif or .eps format.  For more information about figure files please see our guidelines:  https://journals.plos.org/globalpublichealth/s/figures  https://journals.plos.org/globalpublichealth/s/figures#loc-file-requirements | We have provided revised files in tif format according to the guidelines and confirmed through the PLOS NAAS tool that they meet the journal requirements. |
| If the reviewer comments include a recommendation to cite specific previously published works, please review and evaluate these publications to determine whether they are relevant and should be cited. There is no requirement to cite these works unless the editor has indicated otherwise. | Thank you, this is well noted |
| **Additional Editor Comments:** |  |
| Dear authors, Please ensure to upload high resolution graphs in the revision, especially Figure 1 for the model structure. | We have provided revised files in tif format according to the guidelines and confirmed through the PLOS NAAS tool that they meet the journal requirements. |
| **Reviewer 1 Comments:** | |
| This manuscript addresses an important and policy-relevant public health issue: improving measles vaccination coverage in Kenya through economically efficient intervention strategies. The topic is highly relevant to low- and middle-income countries striving to achieve and sustain high immunization coverage amid constrained health budgets. The integration of cost-effectiveness analysis (CEA) and budget impact analysis (BIA) is commendable and provides potential value for immunization policy planning. However, although the study demonstrates promise, several methodological, analytical, and reporting issues should be addressed before the manuscript can be considered for publication | Thank you for the review and feedback. We have now addressed each of the points raised by the reviewers and editors. |
| **Reviewer 2 Comments:** |  |
| **Overall comment:** 1. The study is of great relevance, and should be published, however, minor changes shall be made to improve the quality of the publication. | Thank you. Please see below the changes made. |
| **Methods:** 2. The authors use SIA as a catch-up, they need to clarify this. | We thank the reviewer for this comment. We have better clarified in the manuscript that SIAs were done as catch-up campaigns, conducted every 5 years and targeting all children under 5 years of age, regardless of their prior vaccination status. This approach is consistent with current national practice, whereby SIAs are used to reach children with unknown, incomplete or undocumented vaccination histories and thereby boost overall immunization coverage. |
| 3. Line 94: Describe better the definition of incremental analysis, is it the same as a sensibility analysis? | We thank the reviewer for this comment and for the opportunity to clarify. We have revised the text to better define the incremental cost effectiveness analysis and to distinguish it from the sensitivity analysis, as they are distinct methodological steps in our study.  We have now better clarified that an incremental cost effectiveness analysis was conducted, comparing three vaccination coverage scenarios, each evaluated against the next best alternative to generate incremental cost-effectiveness ratios (ICERS). Specifically, the constant improvement scenario was compared against the base case, and the intensified improvement scenario against the constant improvement scenario. The “cost-effectiveness analysis” subsection in the Methodology later explains in more detail how the ICER is calculated  This main incremental cost-effectiveness analysis is different from the sensitivity analysis, which was subsequently conducted to assess how robust the findings were to uncertainty in key parameters. Although the sensitivity analysis also generates ICERS, based on varying different parameters (e.g. vaccine price, reporting rate), its purpose is to test the robustness of the base incremental results. |
| 4. Line 103, Table 1. Are SIA or catch-up vaccinations every 5 years per life? The authors need to clarify this. | We have now better clarified this in Table 1 to read that SIAs are conducted every 5 years, assuming a 95% coverage to children under 5 years, irrespective of vaccination status. |
| 5. The authors decided to use a Markov model. Markov models are considered static models because they usually assume that the risk of infection remains constant over time and does not change according to how many people are vaccinated. This can be useful for many chronic diseases, but it has important limitations for highly contagious infectious diseases such as measles. For measles vaccination, dynamic models are generally better because they include the effect of transmission between people and the impact of herd immunity. When more individuals are vaccinated, the virus spreads less in the community, indirectly protecting even unvaccinated people. Static Markov models cannot adequately capture this indirect protection. Because measles has a very high transmissibility (R0 of approximately 12–18), small changes in vaccination coverage can dramatically affect outbreak risk. Dynamic models can therefore better estimate the real public health and economic benefits of vaccination, what the authors decided to use a static model? I am not contradicting the methodology of their analysis, but a response to this inquiry is necessary. | We thank the reviewer for this important comment. We agree that dynamic transmission models are generally the preferred approach for evaluating vaccination programs against highly transmissible infections such as measles, as they capture the effect of transmission between people and the indirect effects of vaccination, including herd immunity that static models do not fully capture.  We acknowledge that a dynamic transmission model would have been preferable for this analysis. However, dynamic models also require detailed and reliable data on transmission dynamics, including age-specific contact patterns, population mixing, time-varying transmission rates and other epidemiological parameters, which were not sufficiently available. Given these data limitations, we considered a static Markov model to be an appropriate and pragmatic approach for assessing the direct health and economic effects of alternative measles vaccination strategies using available evidence.  We have acknowledged this limitation in the discussion section of the manuscript. In particular, we note that the Markov model does not fully capture the indirect effects of vaccination, including herd immunity, Consequently, our findings should be interpreted as conservative estimates of the potential health and economic benefits of increasing measles vaccination coverage, as the model may underestimate the overall effectiveness and benefits of vaccination. Future research using a dynamic model, supported by improved epidemiological and contact-pattern data, would be valuable to quantify these indirect effects.  Lastly, given that the Intensified investment scenario aims to achieve 95% coverage for both MCV1 and MCV2, consistent with WHO recommendations for measles elimination and achievement of herd immunity, an additional scenario analysis has been added to explore the potential indirect protection associated with increased population immunity. The infection probability among the unvaccinated individuals was reduced by 25%, 50%, 75% and 82% for the intensified investment scenario. The 82% reduction was informed by Amela Heras et al, who reported a corresponding reduction in measles cases among unvaccinated individuals associated with a vaccination programme. These reductions were exploratory and intended to assess the potential impact of indirect protection. |
| 6. Line 168. Measles’ costs. The authors need to describe in a separate table all related direct and indirect costs (both outpatient and inpatient). | Thank you for pointing this out. We have now included in more detail in the text and Table 1 under the costs of outpatient treatment that these included direct costs to the provider such as consultation costs, laboratory tests, medications, staff costs, utilities and building space. While Inpatient treatment costs included direct costs to the provider such as hospital bed days, staff time, laboratory tests, medications, and other supportive care costs (e.g. blood transfusions, oxygen supplementation, phototherapy and nutrition items).  Indirect costs such as productivity losses related to illness and inability to work (morbidity costs) and lost economic productivity due to death (mortality costs) were excluded because of the health system’s perspective that was adopted. |
| **Results:** 7. The Budget Impact Analysis: Has the authors estimated their data from this analysis to which health interventions that money could be used? | Thank you for pointing this out. Identifying the specific health interventions from which reallocation decisions could be made was beyond the scope of this budget impact analysis, as such decisions are highly contextual and would require further engagement with policy makers. However, these discussions will be had with the relevant stakeholders as we plan to disseminate our findings. |
| **Reviewer 3 Comments:** |  |
| The paper on the cost-effectiveness and budget impact analyses of strategies to improve measles vaccination in Kenya is well-written. The following are my major comments: | Thank you for the feedback. Please see below responses to each comment and changes made |
| 1. The authors are using a static model, rather than a dynamic model. The model does not capture herd immunity and disease transmission effects specific to measles. This is a drawback for a study of measles vaccination. Could the authors explain why they aren't using a dynamic model? Also, did they try to adjust for the uncertainty created in the results from using a static model in their sensitivity analysis? | We thank the reviewer for this important comment. We agree that dynamic transmission models are generally the preferred approach for evaluating vaccination programs against highly transmissible infections such as measles, as they capture the effect of transmission between people and the indirect effects of vaccination, including herd immunity that static models do not fully capture.  However, dynamic models also require detailed and reliable data on transmission dynamics, including age-specific contact patterns, population mixing, time-varying transmission rates and other epidemiological parameters, which were not sufficiently available. Given these data limitations, we considered a static Markov model to be an appropriate and pragmatic approach for assessing the direct health and economic effects of alternative measles vaccination strategies using available evidence.  We have acknowledged this limitation in the discussion section of the manuscript. In particular, we note that the Markov model does not fully capture the indirect effects of vaccination, including herd immunity, Consequently, our findings should be interpreted as conservative estimates of the potential health and economic benefits of increasing measles vaccination coverage, as the model may underestimate the overall effectiveness and benefits of vaccination. Future research using a dynamic model, supported by improved epidemiological and contact-pattern data, would be valuable to quantify these indirect effects.  Lastly, given that the Intensified investment scenario aims to achieve 95% coverage for both MCV1 and MCV2, consistent with WHO recommendations for measles elimination and achievement of herd immunity, an additional scenario analysis has been added to the sensitivity analysis to explore the potential indirect protection associated with increased population immunity. The infection probability among the unvaccinated individuals was reduced by 25%, 50%, 75% and 82% for the intensified investment scenario. The 82% reduction was informed by Amela Heras et al, who reported a corresponding reduction in measles cases among unvaccinated individuals associated with a vaccination programme. These reductions were exploratory and intended to assess the potential impact of indirect protection. |
| 2. In the introduction section, the authors reports the number of measles cases and deaths of 9000 and 165, respectively, between 2003 and 2016. Why aren't more recent data on measles incidence and prevalence used in the text? | Thank you for pointing this out, we have now updated this with more recent data to read as follows:  “In Kenya, an estimated 9,000 confirmed cases and 165 deaths (1.8% case fatality) were reported between 2003 and 2016. More recently, surveillance data indicate approximately 2,900 cases and 18 deaths between January 2024 and February 2025.” |
| 3. The authors assume that the number of measles cases per year are 700 cases. This number seems low since the number of cases reported in 2023 and 2024 were 1,551 and 2,949, respectively. The authors say that they are adjusting for underreporting. Could the authors show the number of cases that are assumed, after adjustment and explain how these are calculated. | Thank you for this query. We agree that the 2023 and 2024 reported cases were high-reflecting outbreaks in those years. For the analysis, given that our model is static, we used the average annual reported cases in Kenya from the WHO Global Health Observatory over 2014-2025 since measles incidence fluctuates substantially between outbreak and non-outbreak years. This gives an average of 700 reported cases per year. We have better clarified this in the text.  We then adjusted for underreporting using a 10% reporting rate (WHO 2012), giving an estimated 7,000 total cases per year in the general population. Of these, we assumed 55% occur in children under 5 (Kisangau 2018), giving 3,850 cases per year in children under 5.  To split the cases by severity, we treated reported and unreported cases separately. For the unreported cases, we assume that a majority (95%) are outpatient cases and only 5% require inpatient. While for the reported cases, 36% require hospitalization (Kisangau 2018). Based on this, overall, 92% of all cases are outpatient (moderate measles) while 8% of cases are inpatient (severe measles) |
| 4. The authors are using vaccination delivery costs taken from another study that are for countries with lower-middle incomes. Why didn't the authors use delivery costs specific to Kenya? | Thank you for this query. We agree that the ideal source would have been primary, Kenyan-specific data on vaccine delivery costs across different coverage levels. However, such disaggregated, coverage-specific delivery cost data were not available for Kenya. We therefore used the study by Levin et al, conducted in settings comparable to Kenya, which modelled how measles vaccine delivery costs change with coverage level based on the intensity of activities needed to reach higher coverage. We considered this a suitable proxy, as it aligned well with the strategies evaluated in our analysis. |
| 5. The authors are using age weighting in their calculation of DALYs which is not commonly done. What is the justification for using age weighting in this calculation? | Thank you for pointing this out. We agree that there has been a long-standing debate on whether to use age-weights in DALY calculations, with more recent studies tending not to incorporate them.  We have adjusted this accordingly, and are still using the standard DALY formula, but with no age weighting applied. We have clarified this in the text under the Disability-adjusted life years sub-section as follows:  “DALYs were calculated considering no age-weighting, a discount rate of 3% and the Kenyan 2024 standard life expectancy (27).”  This is also reflected in our excel based Markov Model where the age-weighting modulating factor(K) in the DALY calculation was set to 0 for no age weights. The results have also been updated. |
| 6. For the budget impact analysis, could the authors indicate whether they are subtracting the cost of averted treatment from the costs of vaccination? Also, what health expenditure are the authors using? Are these government expenditures or do these include all expenditures on health? | Thank you for raising these points.  Regarding the budget impact analysis, the net budget impact is calculated as the total health system cost (treatment and vaccination cost) of each scenario minus the total health system cost of the base case scenario. This explanation has also now been added as a footnote under Table 4.  Regarding the health expenditure data, we use current health expenditure, which aggregates government, external and private expenditures. This was deemed appropriate for assessing affordability, as we apply the standardized budget impact threshold (SBIT) proposed by Pichon-Riviere et al (2025), which is calculated as a proportion of current health expenditure. |
| **Reviewer 4 Comments:** |  |
| The manuscript meets the basic criteria. The data availability which states "All the data relevant to this study are included in the article" is insufficient. There should be a clear indication of where and how the underlying data can be accessed. Authors should justify this with a journal policy if data are restricted. | Thank you for pointing this out. In addition to including all the data relevant in the study within the article, we have also made the Excel-based Markov Model publicly available. This Markov Model includes all the data, formulas and calculations used in the study. We have revised the data availability section to read:  “All data underlying the findings are available within the manuscript, In addition, the Excel-Based Markov Model has been deposited in a public repository and is accessible at: https://doi.org/10.6084/m9.figshare.33164015 ” |
| The authors acknowledged that a static Markov model "does not capture measles disease dynamic transmission effects and herd immunity." This is a significant limitation that needs more prominent treatment. | We thank the reviewer for this important comment. We agree that dynamic transmission models are generally the preferred approach for evaluating vaccination programs against highly transmissible infections such as measles, as they capture the effect of transmission between people and the indirect effects of vaccination, including herd immunity that static models do not fully capture.  However, dynamic models also require detailed and reliable data on transmission dynamics, including age-specific contact patterns, population mixing, time-varying transmission rates and other epidemiological parameters, which were not sufficiently available. Given these data limitations, we considered a static Markov model to be an appropriate and pragmatic approach for assessing the direct health and economic effects of alternative measles vaccination strategies using available evidence.  We have acknowledged this limitation in the discussion section of the manuscript. In particular, we note that the Markov model does not fully capture the indirect effects of vaccination, including herd immunity, Consequently, our findings should be interpreted as conservative estimates of the potential health and economic benefits of increasing measles vaccination coverage, as the model may underestimate the overall effectiveness and benefits of vaccination. Future research using a dynamic model, supported by improved epidemiological and contact-pattern data, would be valuable to quantify these indirect effects.  Lastly, given that the Intensified investment scenario aims to achieve 95% coverage for both MCV1 and MCV2, consistent with WHO recommendations for measles elimination and achievement of herd immunity, an additional scenario analysis has been added to the sensitivity analysis to explore the potential indirect protection associated with increased population immunity. The infection probability among the unvaccinated individuals was reduced by 25%, 50%, 75% and 82% for the intensified investment scenario. The 82% reduction was informed by Amela Heras et al, who reported a corresponding reduction in measles cases among unvaccinated individuals associated with a vaccination programme. These reductions were exploratory and intended to assess the potential impact of indirect protection. |
| The reporting rate (assumed 10%) has a very large effect on ICERs. | Thank you for pointing this out. Yes it did, and therefore we included in the one-way sensitivity changing the reporting rate between 2-20% to see the effect on the ICERs. Additionally, we have now pointed out in the discussion, a recommendation to further improve measles reporting and it reads as follows:  “Our study results were highly sensitive to the measles-case reporting rate. This highlights the need for improving surveillance, and the completeness and accuracy of reported measles cases, which would strengthen measles burden estimates, elimination tracking and future economic evaluations.” |
| Minor typographical and grammatical corrections needed. Page 18 line 278 "USD 3,30000" should be "33000" | This typographical correction has been corrected and updated based on the revised results it now reads:  “For the intensified investment scenario, there is a 100% probability that it would be cost-effective at a USD 4,400 willingness to pay threshold compared to the constant improvement scenario” |
| Figure 3 contains partially obscured ICER labels. | Thank you for this feedback. We have now made the text in Figure 3 easier to read |
| At the references, "African Regio" should be "African Region", "Unites States of America" should be "United States of America | Thank you for pointing this out. This has been corrected appropriately |
|  |  |
| **Figure Resubmissions:**  While revising your submission, we strongly recommend that you use PLOS’s NAAS tool (https://ngplosjournals.pagemajik.ai/artanalysis) to test your figure files. NAAS can convert your figure files to the TIFF file type and meet basic requirements (such as print size, resolution), or provide you with a report on issues that do not meet our requirements and that NAAS cannot fix.  After uploading your figures to PLOS’s NAAS tool - https://ngplosjournals.pagemajik.ai/artanalysis, NAAS will process the files provided and display the results in the "Uploaded Files" section of the page as the processing is complete. If the uploaded figures meet our requirements (or NAAS is able to fix the files to meet our requirements), the figure will be marked as "fixed" above. If NAAS is unable to fix the files, a red "failed" label will appear above. When NAAS has confirmed that the figure files meet our requirements, please download the file via the download option, and include these NAAS processed figure files when submitting your revised manuscript. | We have provided revised files in tif format according to the guidelines and confirmed through the PLOS NAAS tool that they meet the journal requirements. |
