# Supplementary material for: Cost-effectiveness and budget impact analyses of strategies to improve measles vaccination coverage in Kenya
Source: PLOS Glob Public Health. 2026 Sep 22;6(9):e0006440. doi: 10.1371/journal.pgph.0006440 (PMC13596817; doi:10.1371/journal.pgph.0006440)
Supplement: Peer review history

# Peer review: Decision Letter 0

**DOI:** 10.1371/journal.pgph.0006440.r001

**Authors:** Xiao Li

3 Jun 2026

PGPH-D-26-01510

Cost-Effectiveness and Budget Impact Analyses of Strategies to Improve Measles Vaccination Coverage in Kenya

PLOS Global Public Health

Dear Dr. Orangi,

Thank you for submitting your manuscript to PLOS Global Public Health. After careful consideration, we feel that it has merit but does not fully meet PLOS Global Public Health’s publication criteria as it currently stands. Therefore, we invite you to submit a revised version of the manuscript that addresses the points raised during the review process.

Please submit your revised manuscript by Aug 02 2026 11:59PM. If you will need more time than this to complete your revisions, please reply to this message or contact the journal office at globalpubhealth@plos.org. When you're ready to submit your revision, log on to https://www.editorialmanager.com/pgph/ and select the 'Submissions Needing Revision' folder to locate your manuscript file.

Please include the following items when submitting your revised manuscript:

- A letter that responds to each point raised by the editor and reviewer(s). You should upload this letter as a separate file labeled 'Response to Reviewers'.
- A marked-up copy of your manuscript that highlights changes made to the original version. You should upload this as a separate file labeled 'Revised Manuscript with Track Changes'.
- An unmarked version of your revised paper without tracked changes. You should upload this as a separate file labeled 'Manuscript'.

Guidelines for resubmitting your figure files are available below the reviewer comments at the end of this letter.

As the corresponding author, your ORCID iD is verified in the submission system and will appear in the published article. PLOS supports the use of ORCID, and we encourage all coauthors to register for an ORCID iD and use it as well. Please encourage your coauthors to verify their ORCID iD within the submission system before final acceptance, as unverified ORCID iDs will not appear in the published article. *Only*  the individual author can complete the verification step; PLOS staff *cannot*  verify ORCID iDs on behalf of authors.

We look forward to receiving your revised manuscript.

Kind regards,

Xiao Li

Academic Editor

PLOS Global Public Health

**Journal Requirements:**

1. Please note that PLOS Global Public Health has specific guidelines on code sharing for submissions in which author-generated code underpins the findings in the manuscript. In these cases, all author-generated code must be made available without restrictions upon publication of the work. Please review our guidelines at https://journals.plos.org/globalpublichealth/s/materials-and-software-sharing#loc-sharing-code and ensure that your code is shared in a way that follows best practice and facilitates reproducibility and reuse.

2. Please amend your detailed Financial Disclosure statement. This is published with the article. It must therefore be completed in full sentences and contain the exact wording you wish to be published.

i. Please clarify all sources of financial support for your study. List the grants, grant numbers, and organizations that funded your study, including funding received from your institution. Please note that suppliers of material support, including research materials, should be recognized in the Acknowledgements section rather than in the Financial Disclosure.

ii. State the initials, alongside each funding source, of each author to receive each grant. For example: "This work was supported by the National Institutes of Health (####### to AM; ###### to CJ) and the National Science Foundation (###### to AM)."

iii. State what role the funders took in the study. If the funders had no role in your study, please state: “The funders had no role in study design, data collection and analysis, decision to publish, or preparation of the manuscript.”

iv. If any authors received a salary from any of your funders, please state which authors and which funders.

3. We note that your Data Availability Statement is currently as follows: All the data relevant to this study are included in the article.

Please confirm at this time whether or not your submission contains all raw data required to replicate the results of your study. Authors must share the “minimal data set” for their submission. PLOS defines the minimal data set to consist of the data required to replicate all study findings reported in the article, as well as related metadata and methods (https://journals.plos.org/plosone/s/data-availability#loc-minimal-data-set-definition).

For example, authors should submit the following data:

- The values behind the means, standard deviations and other measures reported;

- The values used to build graphs;

- The points extracted from images for analysis.

Authors do not need to submit their entire data set if only a portion of the data was used in the reported study.

If your submission does not contain these data, please either upload them as Supporting Information files or deposit them to a stable, public repository and provide us with the relevant URLs, DOIs, or accession numbers. For a list of recommended repositories, please see https://journals.plos.org/plosone/s/recommended-repositories.

If there are ethical or legal restrictions on sharing a de-identified data set, please explain them in detail (e.g., data contain potentially sensitive information, data are owned by a third-party organization, etc.) and who has imposed them (e.g., an ethics committee). Please also provide contact information for a data access committee, ethics committee, or other institutional body to which data requests may be sent. If data are owned by a third party, please indicate how others may request data access.

4. Please ensure that your Ethics Statement is available in its entirety at the beginning of your Methods section, under a subheading 'Ethics Statement'.

5. We have noticed that you have uploaded Supporting Information files, but you have not included a list of legends. Please add a full list of legends for your Supporting Information files after the references list.

6. Please provide separate figure files in .tif or .eps format.

For more information about figure files please see our guidelines:

https://journals.plos.org/globalpublichealth/s/figures

https://journals.plos.org/globalpublichealth/s/figures#loc-file-requirements

If the reviewer comments include a recommendation to cite specific previously published works, please review and evaluate these publications to determine whether they are relevant and should be cited. There is no requirement to cite these works unless the editor has indicated otherwise.

**Additional Editor Comments (if provided):**

Dear authors,

Please ensure to upload high resolution graphs in the revision, especially Figure 1 for the model structure.

[Note: HTML markup is below. Please do not edit.]

Reviewers' comments:

Reviewer's Responses to Questions

**Comments to the Author**

1. Does this manuscript meet PLOS Global Public Health’s publication criteria?

Reviewer #1: Partly

Reviewer #2: Yes

Reviewer #3: Yes

Reviewer #4: Yes

**********

2. Has the statistical analysis been performed appropriately and rigorously?

Reviewer #1: Yes

Reviewer #2: Yes

Reviewer #3: Yes

Reviewer #4: Yes

**********

3. Have the authors made all data underlying the findings in their manuscript fully available (please refer to the Data Availability Statement at the start of the manuscript PDF file)?

The PLOS Data policy

Reviewer #1: No

Reviewer #2: Yes

Reviewer #3: Yes

Reviewer #4: No

**********

4. Is the manuscript presented in an intelligible fashion and written in standard English?

Reviewer #1: Yes

Reviewer #2: Yes

Reviewer #3: Yes

Reviewer #4: Yes

**********

**Reviewer #1:**  This manuscript addresses an important and policy-relevant public health issue: improving measles vaccination coverage in Kenya through economically efficient intervention strategies. The topic is highly relevant to low- and middle-income countries striving to achieve and sustain high immunization coverage amid constrained health budgets. The integration of cost-effectiveness analysis (CEA) and budget impact analysis (BIA) is commendable and provides potential value for immunization policy planning. However, although the study demonstrates promise, several methodological, analytical, and reporting issues should be addressed before the manuscript can be considered for publication.

**Reviewer #2:**  Overall comment:

The study is of great relevance, and should be published, however, minor changes shall be made to improve the quality of the publication.

Methods:

-The authors use SIA as a catch-up, they need to clarify this.

-Line 94: Describe better the definition of incremental analysis, is it the same as a sensibility analysis?

-Line 103, Table 1. Are SIA or catch-up vaccinations every 5 years per life? The authors need to clarify this.

-The authors decided to use a Markov model.

Markov models are considered static models because they usually assume that the risk of infection remains constant over time and does not change according to how many people are vaccinated. This can be useful for many chronic diseases, but it has important limitations for highly contagious infectious diseases such as measles.

For measles vaccination, dynamic models are generally better because they include the effect of transmission between people and the impact of herd immunity. When more individuals are vaccinated, the virus spreads less in the community, indirectly protecting even unvaccinated people. Static Markov models cannot adequately capture this indirect protection.

Because measles has a very high transmissibility (R0 of approximately 12–18), small changes in vaccination coverage can dramatically affect outbreak risk. Dynamic models can therefore better estimate the real public health and economic benefits of vaccination, what the authors decided to use a static model? I am not contradicting the methodology of their analysis, but a response to this inquiry is necessary.

-Line 168. Measles’ costs. The authors need to describe in a separate table all related direct and indirect costs (both outpatient and inpatient).

Results:

The Budget Impact Analysis: Hae the authors estimated their data from this analysis to which health interventions that money could be used?

**Reviewer #3:**  The paper on the cost-effectiveness and budget impact analyses of strategies to improve measles vaccination in Kenya is well-written. The following are my major comments:

1. The authors are using a static model, rather than a dynamic model. The model does not capture herd immunity and disease transmission effects specific to measles. This is a drawback for a study of measles vaccination. Could the authors explain why they aren't using a dynamic model? Also, did they try to adjust for the uncertainty created in the results from using a static model in their sensitivity analysis?

2. In the introduction section, the authors reports the number of measles cases and deaths of 9000 and 165, respectively, between 2003 and 2016. Why aren't more recent data on measles incidence and prevalence used in the text?

3. The authors assume that the number of measles cases per year are 700 cases. This number seems low since the number of cases reported in 2023 and 2024 were 1,551 and 2,949, respectively. The authors say that they are adjusting for underreporting. Could the authors show the number of cases that are assumed, after adjustment and explain how these are calculated.

4. The authors are using vaccination delivery costs taken from another study that are for countries with lower-middle incomes. Why didn't the authors use delivery costs specific to Kenya?

5. The authors are using age weighting in their calculation of DALYs which is not commonly done. What is the justification for using age weighting in this calculation?

6. For the budget impact analysis, could the authors indicate whether they are subtracting the cost of averted treatment from the costs of vaccination? Also, what health expenditure are the authors using? Are these government expenditures or do these include all expenditures on health?

**Reviewer #4:**  The manuscript meets the basic criteria.

The data availability which states "All the data relevant to this study are included in the article" is insufficient. There should be a clear indication of where and how the underlying data can be accessed. Authors should justify this with a journal policy if data are restricted.

The authors acknowledged that a static Markov model "does not capture measles disease dynamic transmission effects and herd immunity." This is a significant limitation that needs more prominent treatment.

The reporting rate (assumed 10%) has a very large effect on ICERs.

Minor typographical and grammatical corrections needed. Page 18 line 278 "USD 3,30000" should be "33000" figure 3 contains partially obscured ICER labels. At the references, "African Regio" should be "African Region", "Unites States of America" should be "United States of America.

**********

what does this mean?). If published, this will include your full peer review and any attached files.

**Do you want your identity to be public for this peer review?**  If you choose “no”, your identity will remain anonymous but your review may still be made public.

For information about this choice, including consent withdrawal, please see our Privacy Policy

Reviewer #1: No

Reviewer #2: No

Reviewer #3: No

Reviewer #4: **Yes:**  Boahen Derick Atta

**********

[NOTE: If reviewer comments were submitted as an attachment file, they will be attached to this email and accessible via the submission site. Please log into your account, locate the manuscript record, and check for the action link "View Attachments". If this link does not appear, there are no attachment files.]

**Figure Resubmissions:**

# Author comment: Author response to Decision Letter 1

**DOI:** 10.1371/journal.pgph.0006440.r002

12 Aug 2026

Attachment Submitted filename: Response to Reviewers_2026.08.11.docx

# Peer review: Decision Letter 1

**DOI:** 10.1371/journal.pgph.0006440.r003

**Authors:** Julia Robinson

6 Sep 2026

Cost-Effectiveness and Budget Impact Analyses of Strategies to Improve Measles Vaccination Coverage in Kenya

PGPH-D-26-01510R1

Dear Orangi,

We are pleased to inform you that your manuscript 'Cost-Effectiveness and Budget Impact Analyses of Strategies to Improve Measles Vaccination Coverage in Kenya' has been provisionally accepted for publication in PLOS Global Public Health.

Before your manuscript can be formally accepted you will need to complete some formatting changes, which you will receive in a follow up email. A member of our team will be in touch with a set of requests.

Please note that your manuscript will not be scheduled for publication until you have made the required changes, so a swift response is appreciated.

IMPORTANT: The editorial review process is now complete. PLOS will only permit corrections to spelling, formatting or significant scientific errors from this point onwards. Requests for major changes, or any which affect the scientific understanding of your work, will cause delays to the publication date of your manuscript.

If your institution or institutions have a press office, please notify them about your upcoming paper to help maximize its impact. If they'll be preparing press materials, please inform our press team as soon as possible -- no later than 48 hours after receiving the formal acceptance. Your manuscript will remain under strict press embargo until 2 pm Eastern Time on the date of publication. For more information, please contact globalpubhealth@plos.org.

Thank you again for supporting Open Access publishing; we are looking forward to publishing your work in PLOS Global Public Health.

Best regards,

Julia Robinson

Executive Editor

PLOS Global Public Health

***********************************************************

Reviewer Comments (if any, and for reference):

Reviewer's Responses to Questions

**Comments to the Author**

Reviewer #1: All comments have been addressed

Reviewer #2: All comments have been addressed

**********

publication criteria?

Reviewer #1: Yes

Reviewer #2: Yes

**********

3. Has the statistical analysis been performed appropriately and rigorously?

Reviewer #1: No

Reviewer #2: Yes

**********

4. Have the authors made all data underlying the findings in their manuscript fully available (please refer to the Data Availability Statement at the start of the manuscript PDF file)?

The PLOS Data policy

Reviewer #1: Yes

Reviewer #2: Yes

**********

5. Is the manuscript presented in an intelligible fashion and written in standard English?

Reviewer #1: Yes

Reviewer #2: Yes

**********

Reviewer #1: I have carefully reviewed the revised version of the manuscript and the authors' responses to the comments raised during the previous round of review.

The authors have adequately addressed the issues raised in the earlier review and have made appropriate revisions to improve the methodological clarity, presentation, and interpretation of the manuscript. The revised manuscript is clearer and provides an improved presentation of the cost-effectiveness and budget impact analyses of strategies aimed at improving measles vaccination coverage in Kenya.

The analytical approach is appropriate for the stated objectives, and the statistical and economic analyses are presented in a manner that supports the principal findings. The assumptions and interpretation are sufficiently described, and the conclusions are generally consistent with the results presented.

The manuscript addresses an important public health and health-economic question with direct relevance to vaccination policy and resource allocation in Kenya and other settings where measles vaccination coverage remains suboptimal.

Overall, I consider that the authors have satisfactorily responded to the previous review comments and that the manuscript has reached a publishable standard.

Reviewer #2: The authors have satisfactory responded and addressed to all my inquiries.

**********

what does this mean?). If published, this will include your full peer review and any attached files.

**Do you want your identity to be public for this peer review?**  If you choose “no”, your identity will remain anonymous but your review may still be made public.

For information about this choice, including consent withdrawal, please see our Privacy Policy

Reviewer #1: No

Reviewer #2: No

**********
